# Preliminary Findings of the Efficacy of Botulinum Toxin in Temporomandibular Disorders: Uncontrolled Pilot Study

**DOI:** 10.3390/life13020345

**Published:** 2023-01-28

**Authors:** José A. Blanco-Rueda, Antonio López-Valverde, Antonio Márquez-Vera, Roberto Méndez-Sánchez, Eva López-García, Nansi López-Valverde

**Affiliations:** 1 Instituto de Investigación Biomédica de Salamanca (IBSAL), University Hospital, 37007 Salamanca, Spain; 2 Instituto de Investigación Biomédica de Salamanca (IBSAL), Department of Surgery, University of Salamanca, 37007 Salamanca, Spain; 3 Department of Nursing and Physiotherapy, University of Salamanca, 37007 Salamanca, Spain; 4 Primary Care, University Hospital “Rio Hortega”, 47012 Valladolid, Spain; 5 Instituto de Investigación Biomédica de Salamanca (IBSAL), Department of Medicine and Medical Specialties, Universidad Alcalá de Henares, 28801 Madrid, Spain

**Keywords:** botox, botulin toxin therapy, temporomandibular disorders, masticatory dysfunction syndrome, pilot study

## Abstract

Temporomandibular disorders are a common pathology affecting up to 70% of the population, with a maximum incidence in young patients. We used a sample of twenty patients recruited in the Maxillofacial Surgery Service of the University Hospital of Salamanca (Spain), who met the inclusion criteria, with unilateral painful symptomatology of more than three months’ duration. All patients were randomly treated by intramuscular and intra-articular injections of botulinum toxin (100 U) in eight predetermined points. Pain symptomatology was assessed by the visual analog scale (VAS) at the different locations, together with joint symptomatology, at baseline and six weeks after treatment. Adverse effects were also evaluated. In 85% of the patients, pain upon oral opening improved and 90% showed improvement in pain upon mastication. A total of 75% of the patients reported improvement in joint clicking/noise. Headaches improved or disappeared in 70% of the patients treated. Despite the limitations of the study and the preliminary results, intramuscular and intra-articular infiltrations with botulinum toxin were effective in the treatment of symptoms associated with temporomandibular disorders (TMDs), with minimal adverse effects.

## 1. Introduction

Temporomandibular disorders (TMDs) is a term that refers to a number of pathological conditions and disorders related to the temporomandibular joint (TMJ) and its associated musculoskeletal structures [1]. This set of disorders has previously been referred to as TMJ dysfunction syndrome, functional TMJ alterations, myofascial pain dysfunction syndrome, and temporomandibular pain dysfunction syndrome [2].

It is a common condition, with signs appearing in up to 60–70% of the population, yet only one in four people report the presence of any clinical symptoms, and only 5% of patients seek treatment [3]. Although it can appear at any age, the maximum incidence is observed in young adults, between 20 and 40 years of age, predominantly in women, in a ratio of 4/1 with respect to men [4].

More than 50% of TMDs manifest as myofascial pain, produced by parafunctional habits, such as clenching or bruxism. Its etiology remains poorly understood, but it is likely to be multifactorial and include anatomical, pathophysiological, psychosocial, environmental, biological, and genetic factors, including pre-disposing, triggering, and perpetuating factors [5]. Pain is the most common symptom, along with joint noises and functional limitation [6,7]; however, 40% of patients have an exponential reduction in symptoms and 50–90% have pain relief after conservative and rehabilitative treatment, such as physiotherapy, occlusal splints, orthodontic treatments, electrotherapy, etc. [8,9].

Therefore, multidisciplinary approaches are the ideal treatment option, always with the primary goal of resolving the patient’s pain and dysfunction; a recent systematic review suggested that a multidisciplinary approach involving physical medicine and rehabilitation physicians along with dentists is mandatory for the proper diagnosis and treatment of postural disorders in patients with TMDs. [10]

Nonsteroidal anti-inflammatory drugs (NSAIDs), benzodiazepines, antiepileptics, and muscle relaxants are the most commonly used drugs in the treatment of acute pain, with NSAIDs being the most commonly used; however, despite the multiple NSAID options available, only naproxen has been shown to be effective in reducing pain [11]. Muscle relaxants may be prescribed along with NSAIDs if there is evidence of a muscle component, and tricyclic antidepressants are reserved for the treatment of chronic pain [12,13]. 

Botulinum toxin (BTX) is a neurotoxin produced by the anaerobic bacterium *Clostridium botulinum* with forty different serotypes and is one of the most potent toxins. Despite being considered lethal for many centuries, it was the first toxin used in the history of medicine [14]. Serotype A (the most studied for therapeutic purposes) is the most commonly used, although serotype B is occasionally used [15].

There is a gap in the scientific literature and only a limited number of studies refer to the efficacy of botulinum toxin type A (Botox) and its promising results in the improvement of painful myofascial symptoms [16,17,18,19,20] and although it is not considered a first-choice treatment for the management of TMDs, it could be a therapeutic option in situations where conventional treatments are ineffective.

This uncontrolled pilot study analyzed, after six weeks, the efficacy of intra-articular and facial muscle injections of botulinum toxin type A (Botox) on pain and joint clicking/noise associated with temporomandibular joint dysfunction pathology in a sample of twenty patients. Adverse side effects following botulinum toxin injection were also evaluated.

## 2. Materials and Methods

### 2.1. Patients

A sample of 20 patients (n = 20), who met the inclusion criteria for the study, were recruited at the Maxillofacial Surgery Department of the University Hospital of Salamanca. All patients agreed to participate in the research and signed the informed consent form. This study was conducted in accordance with the Helsinki Declaration and was approved by the Salamanca Health Area Drug Research Ethics Committee on 26 April 2021, Reference CEIm:PI 2021 04 734 and was registered in Clinicaltrials.gov (Identifier: NCT05651256).

### 2.2. Patient Description; Inclusion and Exclusion Criteria

The study included patients diagnosed with TMDs, according to the established diagnostic criteria [1], aged between 18 and 69 years (both included) and with unilateral painful symptomatology of more than three months’ duration. Patients previously treated with surgery/arthrocentesis of the TMJ; patients treated in the last six months with surgery in the cervicofacial region; patients who, at the time of inclusion in the study, were being treated in a “Pain Unit”; and patients who had previously received treatment with BTX were excluded. The mean age of the patients was 42.5 years and the distribution by sex was mostly in favor of women, at 17 of the 20 included (85%).

### 2.3. Treatments

**Figure 1:**
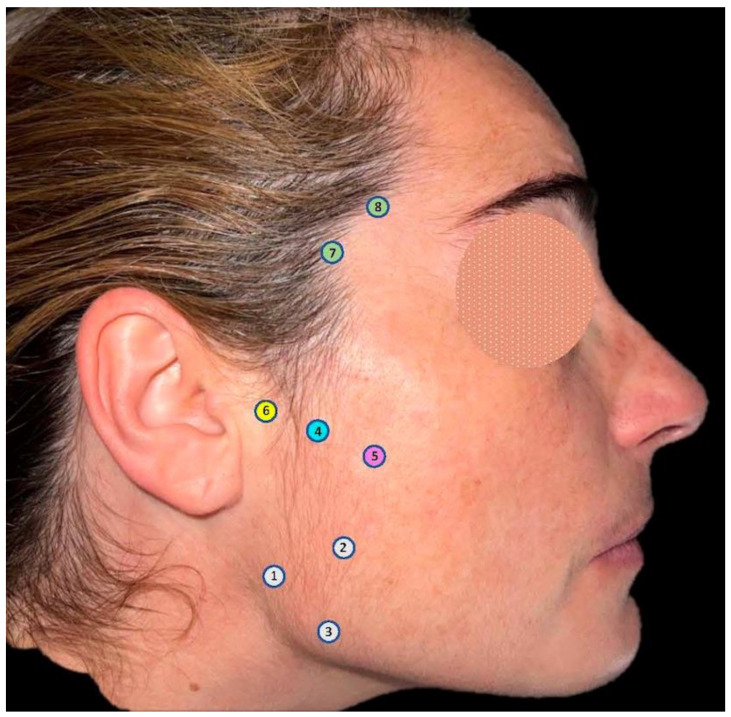
Injection points. 1, 2 and 3, masseter muscle; 4, lateral pterygoid muscle (extra-oral injection); 5, lateral pterygoid muscle (intra-oral injection); 6, TMJ; 7 and 8, anterior temporalis muscle fibers.

The solution for injection was prepared immediately before the intervention, by dissolving the vials of BTX (Botox^®^ 100 U, Allergan Pharmaceuticals, Westport, Ireland), kept refrigerated at 5 °C, in 1 mL of sterile saline solution at room temperature. Eight injection sites were marked: three located in the masseter muscle, two in the lateral pterygoid muscle, one in the TMJ and two in the temporalis muscle (Figure 1).

**Figure 2:**
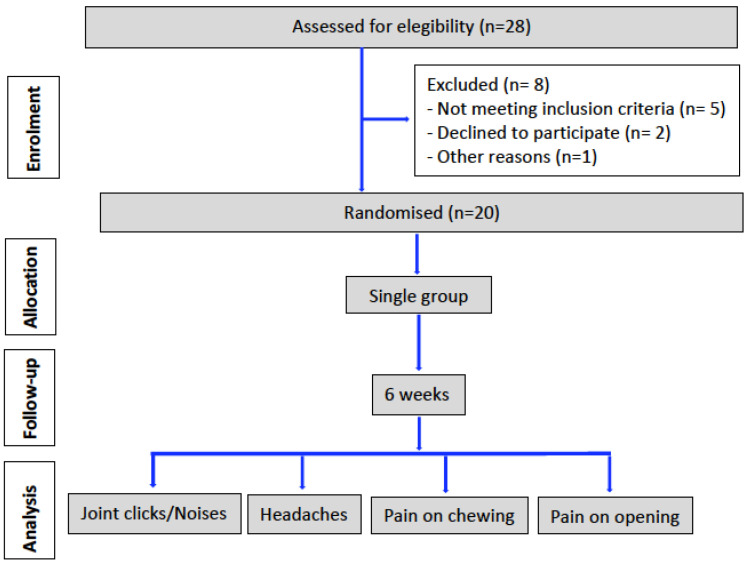
Flow chart of progress through the phases of the study according to the CONSORT statement 2010 [26].

All patients included in the study (Figure 2) were randomized to receive a single dose at each injection site of the prepared solution by a single experienced blinded surgeon, 11 patients (55%) on the right side and 9 (45%) on the left side.

A 1 cc marked insulin syringe was used for intramuscular injection of the prepared solution, according to the locations and amounts proposed by Kim et al. and Ho et al. [21,22], with a total dose of 100 U in each patient, distributed at the different injection sites: 40 U in the masseter muscle, (0.1 cc = 10 U), 20 U in the area of greatest hypertrophy (anterior inferior masseter, point 1), 10 U in the direction of the mandibular inferior border (point 2, middle inferior masseter) and 10 U in the area of the posterior inferior masseter (point 3); 20 U in the lateral pterygoid muscle (10 U extraorally between the zygomatic arch and sigmoid notch and 10 U intraorally, behind the maxillary tuberosity) (points 4 and 5); 20 U in the TMJ (point 6), 10mm anterior to the tragus and 2mm below the zygomatic arch; and 20 U in the anterior part of the temporalis muscle (points 7 and 8). The location of the injection point in the lateral pterygoid muscle was performed taking as a reference, in front of the coronoid process, behind the mandibular condyle, above the zygomatic arch and below the mandibular sigmoid notch, penetrating this area with the needle in an anteroposterior and cranial direction, with an angle of 15° with respect to the anterior border of the mandibular condyle; the injection needle in our study reached a depth of 2.5 cm ± 0.5 cm [23,24,25].

### 2.4. Pain Measurement

Pain was evaluated in different locations of the craniofacial region (temporalis muscle, masseter muscle, pterygoid muscle, trapezius muscle, externocleidomastoid muscle and temporomandibular joint), before BTX treatment and after six weeks, using the visual analog scale (VAS), in a range from 0 to 10, considering a value of 0 as no pain and 10 as maximum pain.

### 2.5. Adverse Effect Assessment

Side effects evaluated included warmth, flushing and hematoma at the injection site, swallowing alteration, contralateral muscle contracture or pain and presence of abnormal jaw movements.

## 3. Results

### 3.1. Pain Reduction

**Figure 3:**
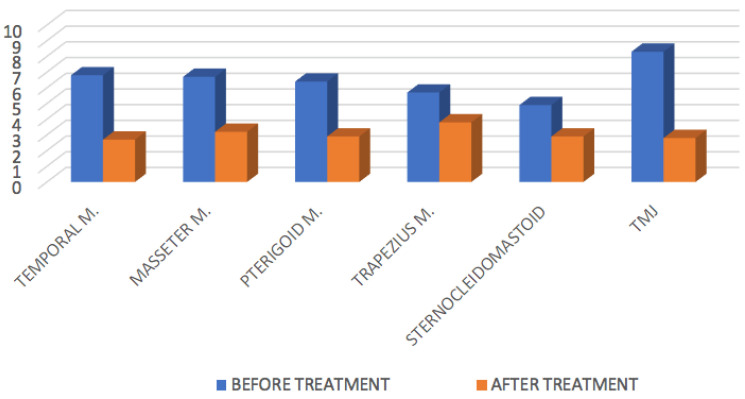
Assessment of pain intensity in the cervicofacial musculature by VAS before and after intramuscular injection of BTX.

The average values of pain intensity, in the different locations, before and after treatment, respectively, are presented in Figure 3.

Before treatment, 19 patients (95%) presented with joint clicking/noise, 17 patients (85%) with headaches, 19 patients (95%) with pain upon opening the mouth and 18 (90%) with pain upon chewing.

At six weeks after treatment, 75% of the patients reported an improvement in joint clicks/noises. Regarding headaches, 70% showed an improvement/disappearance, and only 1 patient (5%) showed a very slight improvement. Eighty-five percent of the patients showed improvement in pain on headaches and 90% showed improvement in pain on chewing. Pain intensity decreased in all areas injected with BTX; however, three patients (15%) reported minimal or no decrease in pain intensity in the different locations.

### 3.2. Adverse Events

It is noteworthy that most of the patients did not report having suffered any adverse event after treatment.

The most frequent adverse effect was the appearance of abnormal jaw movements, which was present in four patients (20%); however, two patients (10%) reported that these abnormal movements were only present in the first few days after treatment, while two others reported that they continued to suffer them.

**Figure 4:**
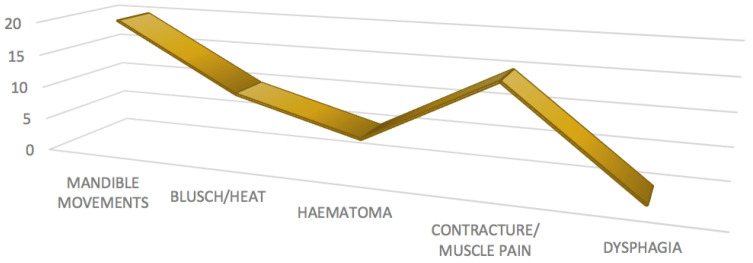
Adverse effects after BTX treatment.

Two of the patients (10%) reported a sensation of flushing/heat in the injection area in the days following treatment, and only one of the patients (5%) reported the appearance of hematoma in the injected area. Three patients (15%) reported the appearance of muscle contracture/pain on the side contralateral to the injection; however, it is noteworthy that swallowing alteration did not appear in any of the patients (Figure 4).

## 4. Discussion

Botulinum toxin has evolved from being a toxicant to a versatile therapeutic tool for a growing list of pathologies; however, treatment with BTX remains controversial and there are not many studies evaluating its efficacy in the treatment of TMDs, most of them being in favor of its use and its ability to alleviate pain associated with this pathology [27,28,29,30].

Freund et al. in 1999 [31] were the first to report preliminary results on the benefits of BTX on pain, function and mouth opening in a sample of 15 patients diagnosed with TMDs. Subsequently, these same investigators, in an expanded sample of 46 patients, demonstrated that intramuscular injections of BTX-A produced significant improvements in pain, function and mouth opening, reducing the severity of symptoms and improving the functional abilities of patients with TMDs, and that these effects would extend beyond its capacity as a muscle relaxant [32]. Recently, several studies and systematic reviews have focused on the benefits of BTX in patients with TMDs [33,34,35,36,37], although there are discrepancies regarding the efficacy of BTX in this pathology, mainly based on methodological errors. Laskin pointed out that many studies do not address the etiology and focus only on symptomatic treatment and that conservative treatments would be equally effective; however, he acknowledges that the etiology of pain and masticatory muscle dysfunction remains unknown [10,38].

Preliminary results of our study found a beneficial effect in 85% of patients with TMDs. These results coincided with those obtained by Sidebottom et al. [27] who, in a prospective study on 62 patients, with a mean age of 42.5 years and (like our study) a preponderance in the sample of the female sex, reported a significant reduction in pain, greater than 90% in 79% of patients treated with a single dose of BTX, concluding that intramuscular injections of BTX, although not guaranteeing complete resolution of myofascial pain, often produce a beneficial effect on symptomatology, and should be considered as an alternative treatment for masticatory myofascial pain in the face of failure of conservative treatments.

The facial areas injected with BTX differ in different studies. Some inject only the masseter muscle [39], others the masseter and temporalis [40,41] and others use injection in the temporalis, masseter, and external pterygoid muscles [42]. Intramuscular injection with BTX in the masseter muscle is often used in the treatment of myofascial syndrome, whose etiological factors lie mainly in fatigue or spasm of the masticatory muscles, with a concomitant arthralgia of the TMJ [27,43]. Schwartz and Freund base the diagnosis of TMDs on two etiologies, one anatomical and the other functional, with two critical areas, the arthrogenic pathology (intracapsular) and the myogenic pathology, originating in the musculature, recommending their simultaneous approach [44]; however, Batifol [45] recommends intra-articular injections exclusively if the pain is chronic. In accordance with these recommendations, our study included patients with a history of pain lasting more than 3 months.

Although BTX was initially only used in the treatment of focal dystonia, it has been shown to provide relief from migraine and tension headaches and neck pain (cervical dystonia), suggesting a possible role in the treatment of TMDs [46,47,48,49,50]; a review by Ihde and Konstantinovic concluded that BTX appears to be relatively safe and effective in the treatment of cervical dystonia and chronic facial pain associated with masticatory hyperactivity [51]. Similarly, Blitzer and Sulica demonstrated its effectiveness in the treatment of torticollis, including as involved muscles the sternocleidomastoid, trapezius, semispinalis capitis, splenius capitis, levator scapulae, and minor paraspinal muscles [52] and that BTX was the most frequently injected substance, intramuscularly, in the treatment of TMDs [53].

Our study, in accordance with these results, found a disappearance of headaches in 70% of the patients treated, although 15% of the patients reported not having found relief from painful symptomatology. In our study, we combined BTX infiltration in the masseter, temporalis, and pterygoid muscles with intra-articular infiltration, obtaining excellent results on pain in the cervicofacial musculature (Figure 3).

On the other hand, intra-articular infiltration of BTX has demonstrated its efficacy in reducing pain [54]. Lora et al. demonstrated in a study in rats the strong antinociceptive effect of intra-articular injection of BTX-A [55]. Intra-articular injection of BTX inhibits inflammatory mediators, reduces neuropeptide release from joint nociceptors and thus reduces neurogenic inflammation and joint pain [56]. Our study found in 85% of treated patients an improvement of pain on mouth opening and 90% showed improvement of pain on chewing. Furthermore, in 75% of the treated patients, an improvement in joint clicking/noise was observed. These results would agree with those obtained by Bakke et al. [57], who, by BTX-A injections into the lateral pterygoid muscle, temporarily reduced the muscle action, but the clicking was permanently eliminated and did not reappear during the 1-year observation period, obtaining a small but clear positional improvement in the disc–condyle relationship. Similarly, other studies [58,59,60] with BTX-A injections into the lateral pterygoid muscle reported benefits on temporomandibular clicking.

The dose ranges are also discrepant in the different studies. Schwartz and Freund recommend doses like those used by us: 25–50 U in the masseter muscle, 5–25 U in the temporalis muscle and 5–25 in the pterygoid muscle [44]. Batifol recommends for intra-articular injection doses similar to those used in our study [45].

Regarding adverse effects, Ihde and Konstantinovic, in a review identifying randomized clinical trials evaluating patients treated with botulinum toxin, found that the adverse effects were mild and transient [61]; similarly, a systematic review by Machado et al. [62] found no significant difference in adverse effects between BTX-A and a placebo. In our study, only four patients (20%) reported them, reporting their disappearance within a few days.

Despite these results, we are aware of the limitations of the study: on the one hand, the small sample size, although authors such as Sipahi Calis et al. and Patel et al. [42,63] base their studies of the effect of BTX on TMDs on sample sizes similar to ours, at 25 and 20 patients, respectively; on the other hand, the evaluation was performed six weeks after the infiltrations, clearly resulting in a reduced evaluation time. In addition, joint and muscle infiltrations, at the same time, could lead to biases in the results. Nevertheless, we present a pilot study with preliminary results.

## 5. Conclusions

Despite the limitations of the study and presenting preliminary results, we believe that multiple intramuscular and intra-articular infiltrations with BTX-A, with a total dose of 100 U, are effective (at least temporarily) in relieving TMJ pain and clicking and noises, with minimal adverse effects. Future ongoing controlled studies, with a larger sample of patients and longer-term follow-up, will allow comparison of the results.

## Data Availability

https://beta.clinicaltrials.gov/.
